# Optical Coherence Tomography Angiography Findings After Intravitreal Ranibizumab in Patients With Coats Disease

**DOI:** 10.3389/fmed.2020.615015

**Published:** 2021-01-21

**Authors:** Gilda Cennamo, Daniela Montorio, Chiara Comune, Maria Paola Laezza, Matteo Fallico, Maria Elena Lionetti, Michele Reibaldi

**Affiliations:** ^1^Department of Public Health, University of Naples Federico II, Naples, Italy; ^2^Department of Neurosciences, Reproductive Sciences and Dentistry, University of Naples Federico II, Naples, Italy; ^3^Department of Ophthalmology, University of Catania, Catania, Italy; ^4^Department of Pediatrics, Università Politecnica delle Marche, Ancona, Italy; ^5^Department of Surgical Sciences, University of Torino, Turin, Italy

**Keywords:** SD-OCT, OCTA, Coats disease, anti-VEGF injections, retinal vascular features

## Abstract

The aim of this retrospective study was to describe the vascular features in eyes with Coats disease, using optical coherence tomography angiography (OCTA), at baseline and after 3 monthly intravitreal injections of ranibizumab. Fifteen eyes of 15 consecutive patients affected by Coats' disease were recruited in this study. All patients underwent the best-corrected visual acuity (BCVA) evaluation, fundus examination, fluorescein angiography (FA), indocyanine green angiography (ICGA), multicolor imaging, structural Spectral Domain (SD)-OCT and OCTA at baseline and 1 month after the third monthly ranibizumab injection (loading phase). Fifteen patients completed the study, of whom nine were males and six females. Mean age was 20.4 ± 2 years. BCVA was 0.46 ± 0.11 logMar and 0.47 ± 0.12 logMar at baseline and after treatment, respectively (*p* = 0.164). SD-OCT revealed no significant decrease in central macular thickness (486.33 μm ± 93.37 at baseline vs. 483.4 μm ± 80.97 after treatment; *p* = 0.915). The subretinal exudates persisted in macular region after intravitreal injections. OCTA showed a general vascular rarefaction in superficial capillary plexus (SCP), deep capillary plexus (DCP), and choriocapillary (CC) that did not change after loading phase. This study showed no functional and vascular improvement following 3 monthly ranibizumab injections. OCTA, non-invasive technique, could be useful during follow up of these patients and provide a better understand of pathogenesis of this disorder.

## Introduction

Coats disease is an idiopathic retinal vascular disorder, described for the first time by Coats in 1908 (1).

This disease is characterized by retinal telangiactasias in the temporal-macular zone, numerous yellowish exudates in the subretinal space containing cholesterol crystals, macular edema, hemorrhages and, in advanced end-stage, by exudative retinal detachment with secondary neovascular glaucoma (2).

The use of intravitreal anti-vascular endothelial growth factor (VEGF) agents in combination with common treatments, such as laser photocoagulation, has been reported to reduce the subretinal fluid (SRF) and exudation in small case series of Coats disease (3–5).

The gold standard test for diagnosis and follow-up after treatment for Coats patients is fluorescein angiography (FA), while optical coherence tomography (OCT) allows to identify macular edema and exudates in macular region (6).

OCT Angiography (OCTA) is a non-invasive imaging technique that provides a detailed evaluation of retinal and choriocapillaris (CC) microvasculature and it turned to be useful and safe in pediatric patients because it does not need intravenous dye agent injection (7, 8).

Previous reports focused on the retinal and CC vascular alterations at OCTA in patients with Coats disease rather than to analyze the vascular changes after VEGF injections (9–13).

The aim of this study was to investigate the structural and vascular features in retina and choriocapillaris at Spectral Domain (SD)-OCT and OCTA in 15 patients affected by Coats disease undergoing anti-VEGF injections.

## Materials and Methods

Fifteen patients affected by Coats disease were retrospectively recruited in this observational study from January 2016 to January 2019 at the Eye Clinic of the University of Naples “Federico II.”

The diagnosis of Coats disease was based on the presence of idiopathic retinal telangiectasia, hemorrhagic phenomena, intraretinal and/or subretinal exudation at fundus examination (2).

Ten patients presented the stage 2A (telangiectasia and extrafoveal exudation), five patients showed the stage 2B (telangiectasia and foveal exudation) in according to the clinical classification introduced by Shields et al. (14).

Exclusion criteria included: vitreoretinal diseases secondary to other causes, congenital retinal disorders, history of intraocular surgery, trauma, and previous treatments before ranibizumab (such as laser photocoagulation, cryotherapy, and other anti-VEGF intravitreal injections).

At baseline all patients underwent a complete ophthalmological examination, including the best-corrected visual acuity (BCVA) evaluation, fundus examination, fluorescein angiography (FA), indocyanine green angiography (ICGA), multicolor imaging (Spectralis, Heidelberg Engineering, Heidelberg, Germany), structural SD-OCT and OCTA (RTVue XR Avanti, Optovue, Inc., Freemont, California, USA).

These patients underwent 3 monthly intravitreal injections of ranibizumab (0.5 mg/0.05 ml) (loading phase) with a 30-gauge needle through the pars plana under aseptic conditions. One month after loading phase (LP) they underwent the measurement of BCVA, intraocular pressure, fundus examination, SD-OCT and OCTA to evaluate the central macular thickness (CMT) and the retinal and choriocapillaris vascular networks, respectively.

The study was registered in https://clinicaltrials.gov/ (NCT04310631) and all investigations adhered to the tenets of the Declaration of Helsinki. Written informed consents were obtained from the patients enrolled in the study.

### OCT-Angiography

OCTA images was acquired using XR Avanti AngioVue OCTA (software ReVue version 2017.1.0.151, Optovue Inc., Fremont, CA, USA). It is a device with a high speed of 70,000 axial scans per second that uses a light source of 840 nm and an axial resolution of 5 μm (15).

The OCT-A software visualized the macular capillary network in scans centered on the fovea by performing a 6 × 6 mm scan and it analyzed the superficial capillary plexus (SCP), deep capillary plexus (DCP) and CC. The superficial vascular plexus was selected at a 60 μm thickness from the inner limiting membrane to include all the vessels of this plexus. A 30 μm thick layer from the inner plexiform layer visualized the entire deep retinal plexus. For the choriocapillaris, the inner and outer boundaries were set at 31 and 59 μm beneath the retinal pigment epithelium (RPE) reference line, respectively (16).

The images with a signal strength index <80, visible eye motion, blinking artifacts and low-quality images obtained with OCT and OCTA did not considered in this analysis.

### Statistical Analysis

Statistical analysis was performed with the Statistical Package for Social Sciences (Version 20.0 for Windows; SPSS Inc, Chicago, Ill, USA). The paired Student's test was used to evaluate the differences in CMT and BCVA between the baseline and after 3 monthly intravitreal injections of ranibizumab. A *p*-value of < 0.05 was considered statistically significant.

## Results

A total of 15 patients affected by unilateral Coats disease (nine males and six females, mean age 20.4 ± 2 years) was included in this retrospective study. Demographic and clinical information of patients were documented in Table 1.

**Table 1:** Demographic, ophthalmologic characteristics and SD-OCT parameters in patients with Coats disease before and after loading phase of intravitreal injections of ranibizumab.

	**Baseline**	**After LP**	***P*-value**
Eyes n.	15	–	–
Sex (male/female)	9/6	–	–
Age (years; mean ± SD)	20.4 ± 2	–	–
Disease stage			
2A	10	–	–
2B	5	–	–
IOP (mmHg)	14 ± 2.03	13 ± 1.2	0.186
BCVA (logMAR)	0.46 ± 0.11	0.47 ± 0.12	0.164
CMT, μm	486.33 ± 93.37	483.4 ± 80.97	0.915

*Data expressed as mean ± SD*.

*SD, Standard Deviation; LP, Loading Phase; IOP, Intraocular Pressure; BCVA, Best Corrected Visual Acuity; logMAR, logarithm of the minimum angle of resolution; CMT, Central Macular Thickness*.

*The paired Student's test, p < 0.05*.

The patients presented a reduced BCVA (0.46 ± 0.11 logMar) and the intraocular pressure was normal (mean 14 ± 2.03 mmHg). At SD-OCT the presence of intraretinal and subretinal exudates and SRF in macular region caused a significant structural alterations mainly in outer retinal layers (ellipsoid zone and external limiting membrane distruption).

After LP, there were no functional and morphological improvements. The patients did not show any change in BCVA respect to baseline (0.47 ± 0.12 logMar vs. 0.46 ± 0.11 logMar, *p* = 0.164), as well as in IOP values (14 ± 2.03 mmHg vs. 13 ± 1.2 mmHg, *p* = 0.186) and in lens transparency.

SD-OCT revealed no significant reduction in CMT (486.33 μm ± 93.37 at baseline vs. 483.4 μm ± 80.97 after LP; *p* = 0.915). Moreover, the presence of the subretinal and intraretinal exudates persisted in macular region after intravitreal injections.

At OCTA in all cases before treatment, the superficial capillary plexus showed few areas of capillary hypoperfusion with a slight increased FAZ area. Irregularly dilatated small perifoveal vessels and an increased vascular rarefaction with loss of the some of their collateral branches were visible in deep capillary network. The CC was partially obscured due to the presence of subretinal exudates that caused a posterior shadow effect.

**Figure 1:**
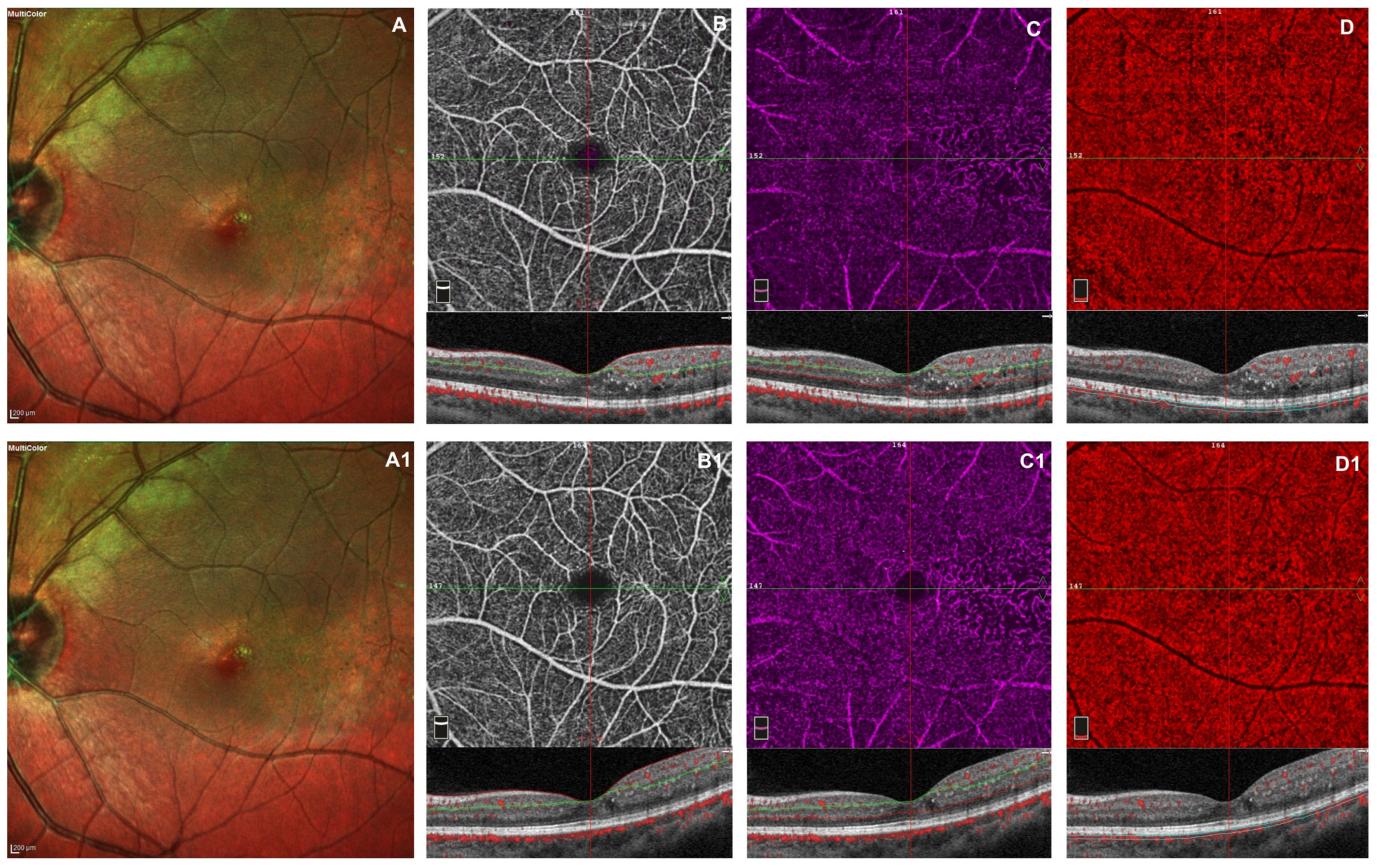
Left eye of a male 20 years old patients before and after 3 monthly intravitreal injection of ranibizumab. At baseline, multicolor shows foveal exudates and retinal telangiectasia temporally to the fovea **(A)**. OCTA images of the superficial capillary plexus (SCP) shows few areas of capillary hypoperfusion with a slight increased FAZ area **(B)**. The deep capillary plexus (DCP) **(C)** shows irregularly dilatated small vessels, increased vascular rarefaction, in particular temporally the fovea. The CC reveals few areas of no flow signal due to a posterior shadow effect from intraretinal exudates **(D)**. The structural spectral domain optical coherence tomography (SD-OCT) B-scan of each OCTAngiography (OCTA) image reveals some intraretinal exudates and cysts temporally the foveal region. After loading phase, the multicolor image reveals no change in foveal exudates and in teleangectasia temporally to the foveal region **(A1)**. At OCTA no significant improvement in capillary drop out and anomalies in vessel size are found in superficial and deep capillary networks, respectively **(B1,C1)**. In CC persists few areas of no flow signal due to the presence of intraretinal exudates **(D1)**. No structural OCT changes was evident after treatment.

The retinal and choriocapillaris vascular impairment persisted after VEGF injections in all patients revealing no improving in capillary drop out and anomalies in vessel size in superficial and deep capillary networks. The vascular rarefaction of CC remains evident due to the persistent presence of intraretinal exudates (Figure 1).

## Discussion

Coats disease is a retinopathy characterized by idiopathic vascular abnormalities in retinal periphery, subretinal and intraretinal extensive exudation (18). Exudates contains cholesterol crystals and tends to affect the macula with SRF accumulation and macular edema (12). When untreated, Coats disease leads to vision loss due to exudative retinal detachment, and less frequently neovascular glaucoma (12). Many treatments have been used for this disease in relation to the stage. Initial treatment includes ablation therapy with cryotherapy or laser photocoagulation of the telangiectasic areas, but SRF and massive exudation are often responsible for therapeutic failure (18). In last years, some studies have demonstrated good therapeutic results using intravitreal anti-VEGF injections (18–20). Some authors have found high levels of VEGF in aqueous and vitreous of eyes affected by Coats disease (21–23). Moreover, Yang et al. (18) demonstrated that the intravitreal ranibizumab injections (IVR) may reduce the SRF, exudation and hyperpermeability of telangiectasia in stages 3A and 3B Coats disease, improving the visual acuity. In particular, the authors treated 17 eyes with monthly IVR in the first 3 months as an initial treatment, which was then combined with another ablative therapy, such as laser photocoagulation or cryotherapy (18). Conversely, Li et al. (24) reported the improvement of the visual acuity after IVR only in cases without exudation of cholesterol crystals under the macular area. The study conducted by Chiu et al. (19) showed a functional recovery in 30 patients, even if the worse visual outcomes occurred for more advanced stages of the disease.

Moreover, OCTA, allowed to detect the retinal and CC vascular abnormalities in Coats disease (10, 13) also in patients after laser treatment and cryotherapy (12).

In this study we found areas of capillary rarefaction in superficial and deep retinal plexuses and in CC and we didn't note any vascular and structural changes after IVR therapy. According to Sen et al., we believed that intravitreal anti-VEGF injections does not represent the treatment of Coats disease, because the increased levels of VEGF, found in this disease, are not involved in its pathogenesis (6). Indeed, the destruction of the blood-retinal barrier, due to alterations of the endothelial cells, causes plasma and exudates leakage into the retina. Moreover, the presence of abnormal pericytes and the damaged epithelium determine the formation of telangiectasia and the closure of the vessels with consequent retinal ischemia (6, 17). These structural vascular changes rather than the increase of VEGF levels would explain the reason of the absence of structural and functional improvements after anti-VEGF therapy, found in our results.

The study presents several limitations, especially the relatively small sample size of the groups, the retrospective nature, the short follow up and absence of quantitative evaluation of retinal and CC microvasculature. Moreover, the analysis of OCTA at posterior pole turned to be influenced by the presence of the intraretinal exudates that caused, due to the masking effect, a reduced visualization of the vascular network of CC.

In conclusion, in this study OCTA is a non-invasive technique, useful in the diagnosis and follow up of this retinopathy and allow to better understand the pathophysiology of this disease.

Further longitudinal studies and a longer follow up are needed to analyze a larger number of patients affected by this rare disease.

## Data Availability Statement

The original contributions presented in the study are included in the article/supplementary material, further inquiries can be directed to the corresponding author.

## Ethics Statement

The studies involving human participants were reviewed and approved by Clinical trials. The patients/participants provided their written informed consent to participate in this study.

## Author Contributions

GC conceived and designed the study. DM and MPL performed data collection. DM analyzed data and designed the statistical analysis. DM, CC, and MPL wrote the manuscript. GC, MF, and MEL revised the manuscript. GC and MR undertook supervision. All authors have read and agreed to the published version of the manuscript.

## Conflict of Interest

The authors declare that the research was conducted in the absence of any commercial or financial relationships that could be construed as a potential conflict of interest.
